# Impact of Metabolic Dysfunction-Associated Steatotic Liver Disease on Fatigue and Pruritus in Primary Sclerosing Cholangitis: A U.S. Single-Center Study

**DOI:** 10.3390/jcm14228083

**Published:** 2025-11-14

**Authors:** Natalia Rojas-Amaris, Ana Marenco-Flores, Carmen Lara-Romero, Romelia Barba, Denisse Rubio-Cruz, Ximena Parraga, Daniela Goyes, John Esli Medina-Morales, Leandro Sierra, Manuel Romero-Gomez, Michelle Lai, Behnam Saberi, Vilas Patwardhan, Alan Bonder

**Affiliations:** 1 Division of Gastroenterology, Hepatology, and Nutrition, Beth Israel Deaconess Medical Center, Harvard Medical School, Boston, MA 02215, USA; nrojasam@bidmc.harvard.edu (N.R.-A.); vpatward@bidmc.harvard.edu (V.P.); 2 UCM Digestive Diseases Unit, Virgen del Rocío University Hospital, Institute of Biomedicine of Seville, CIBEREHD, University of Sevilla, 41013 Sevilla, Spain; 3 Department of Internal Medicine, Texas Tech University System, Lubbock, TX 79430, USA; 4 Division of Digestive Diseases, Yale School of Medicine, New Haven, CT 06520, USA; 5 Division of Gastroenterology, Washington University School of Medicine, St. Louis, MO 63110, USA; 6 Department of Medicine, Cleveland Clinic Foundation, Cleveland, OH 44195, USA

**Keywords:** primary sclerosing cholangitis, MASLD, patient-reported outcomes, fatigue, pruritus, itching

## Abstract

**Background and Aims:** Metabolic dysfunction-associated steatotic liver disease (MASLD) is the most common cause of liver disease in the United States and frequently coexists with other liver diseases. Despite growing interest, the presence of MASLD in patients with primary sclerosing cholangitis (PSC) remains underexplored. This study aimed to assess the prevalence and characteristics of the MASLD-PSC overlap syndrome, with a specific focus on patient-reported outcomes such as pruritus and fatigue. **Methods:** A cross-sectional analysis was performed within a prospective cohort of patients with PSC enrolled in the Autoimmune Liver Diseases Registry at a United States tertiary medical center (2018–2024). MASLD overlap was established based on evidence of hepatic steatosis on liver imaging or biopsy, combined with at least one cardiometabolic risk factor. Fatigue and pruritus were assessed using the Chronic Liver Disease Questionnaire (CLDQ) and the 5D Itch Scale. Ordinal logistic regression models were used to explore the potential impact of MASLD overlap on fatigue and pruritus severity. **Results:** Among 103 PSC patients, 33% had MASLD overlap. These patients were older (55 vs. 46 years, *p* = 0.006), had a higher BMI (30 vs. 25 kg/m^2^, *p* < 0.001), and were more likely to have small bile duct involvement (43% vs. 12%, *p* = 0.002). A history of liver transplantation (LT) was noted in 18% of PSC-only patients, compared to 3% of those with PSC/MASLD (*p* = 0.055). MASLD overlap was significantly associated with higher pruritus intensity (OR 3.09, 95% CI 1.02–9.28, *p* = 0.044), but was paradoxically linked to lower fatigue levels (OR 0.37, 95% CI 0.16–0.85, *p* = 0.020). **Conclusions:** Patients with PSC/MASLD exhibit distinct clinical features. MASLD overlap was found to significantly impact patient-reported outcomes, with lower fatigue intensity but increased pruritus severity, suggesting a role for metabolic or inflammatory pathways, warranting further investigation.

## 1. Introduction

Metabolic dysfunction-associated steatotic liver disease (MASLD) is the most common chronic liver disease worldwide, affecting over one-third of U.S. adults [1,2]. The condition ranges from simple steatosis to metabolic dysfunction-associated steatohepatitis (MASH), which can progress to fibrosis or cirrhosis [3]. Recent analyses from the Global Burden of Disease studies show a quickly increasing health and economic impact [4]. A meta-analysis estimated the prevalence of MASLD at 38% globally (44.4% in Latin America, 25.1% in Western Europe) and projects an increase to 55.4% by 2040 [5]. Primary sclerosing cholangitis (PSC) is a rare, chronic cholestatic liver disease characterized by inflammation and fibrosis of the bile ducts [6,7]. Previous cohort studies have found MASLD and PSC overlap in 8–14.7% of PSC cases, complicating management, worsening symptoms, and increasing the risk of complications and mortality [8,9].

Patient experiences highlight the clinical challenges of chronic liver disease. Individuals with these conditions often describe their illness as a fluctuating experience marked by uncertainty about prognosis, ongoing vulnerability, and perceived effects on work, social roles, and personal identity [10,11]. Qualitative studies document physical symptoms, psychological distress, and reduced social functioning that together lower quality of life [12].

In PSC, pruritus and fatigue are particularly disabling; severe pruritus is associated with more advanced disease and poorer quality of life [13]. Among adults with metabolic dysfunction-associated steatotic liver disease (MASLD), 37% report significant pruritus and 33% report persistent fatigue [14]. These symptoms often occur with depression, anxiety, sleep disturbances, and social isolation, and they contribute to decreased well-being and lower work productivity [15].

Against this backdrop, challenges increase when patients have more than one chronic liver disease. Managing both a metabolic and a cholestatic condition involves navigating the uncertainty of progressive cholangitis while also treating metabolic comorbidities. Patients living with multiple chronic conditions describe lengthy diagnostic journeys, limited support, and a persistent sense of vulnerability worsened by fragmented care and poor information [11,16]. A high, ongoing symptom burden predicts worse mental health, poorer clinical outcomes, and higher mortality even when laboratory results do not seem severe [17]. A systematic review by Kim et al. found that only 3 of 83 patient-reported outcome (PRO) instruments for cholestatic disease included patient input, emphasizing how rarely patients are asked what matters most to them [18].

The overlap between MASLD and PSC remains poorly understood, with limited data on symptom patterns, health-related quality of life (HRQoL), and clinical outcomes. This study aims to determine the prevalence of MASLD-PSC overlap, compare PROs related to pruritus and fatigue in PSC patients with and without MASLD using validated tools, and explore the relationship between overlap status and clinical outcomes. The goal is to enhance understanding by emphasizing the impact of MASLD-PSC overlap from the patient’s perspective, shifting the focus from epidemiological data to patient-centered care.

## 2. Materials and Methods

### 2.1. Study Population

We conducted a cross-sectional analysis within a prospective cohort study using data from a single visit of individuals with PSC enrolled in the Autoimmune Liver Diseases Registry between January 2018 and October 2024 at Beth Israel Deaconess Medical Center (Boston, MA, USA). PSC diagnosis was established based on the American Association for the Study of Liver Diseases (AASLD) guidelines [19]. MASLD overlap was confirmed by the presence of hepatic steatosis on liver imaging or biopsy, along with at least one cardiometabolic risk factor, while excluding other causes of steatosis according to the Delphi consensus [20].

**Figure 1:**
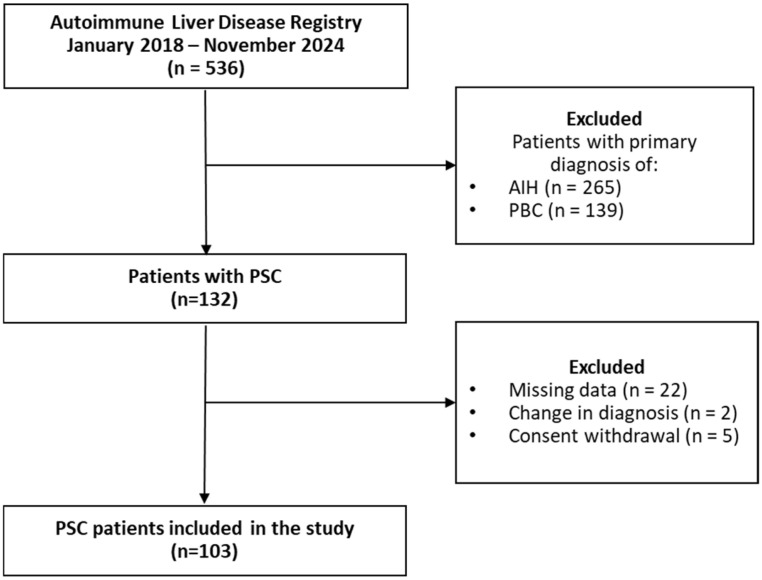
Flowchart of study subjects’ selection. AIH, Autoimmune Hepatitis; *n* number; PBC, primary biliary cholangitis; PSC, primary sclerosing cholangitis.

Among individuals with PSC enrolled in the registry, participants were excluded if they had missing clinical data, including laboratory results, imaging findings, and questionnaire responses. Additional exclusion criteria included withdrawal of consent and changes in primary diagnosis (Figure 1). The final sample consisted of 103 participants with PSC who met the necessary criteria for analysis.

### 2.2. Study Outcome, Variables, and Definitions

The primary outcome of this study was to determine the influence of MASLD overlap on the intensity of fatigue and pruritus reported by patients with PSC. These patient-reported outcomes (PROs) were assessed using the Chronic Liver Disease Questionnaire (CLDQ) and the 5D Itch Scale, which participants completed voluntarily following their liver clinic visits. Trained staff were available to provide assistance as needed. Both fatigue and pruritus severity were measured using the ordinal scales provided by these validated instruments.

The prevalence of MASLD overlap in the PSC population and the evaluation of clinically significant differences between individuals with and without this condition were also considered key secondary outcomes. To achieve this, clinical data were extracted from participants’ electronic medical records using the REDCap (Research Electronic Data Capture) database. Extracted data included demographic information; history of IBD, cirrhosis, cholangiocarcinoma, liver transplant, comorbidities (diabetes mellitus and/or hypertension), bile duct involvement; and liver imaging studies (e.g., FibroScan, or MRI).

Additionally, laboratory values collected comprised alkaline phosphatase (ALP), alanine transaminase (ALT), aspartate aminotransferase (AST), cholesterol (HDL, LDL, and total), triglycerides, creatinine, total bilirubin (TB), albumin (ALB), platelets (PL), white blood cell count (WBC), prothrombin time (PT), and international normalized ratio (INR).

### 2.3. Statistical Analysis

Continuous data were reported as medians with interquartile ranges (IQR), while categorical variables were summarized as percentages. A non-parametric distribution was assumed for all variables given the study’s sample size. Post-hoc power analysis confirmed that the sample size (*n* = 103) provided excellent statistical power (>99%) for both primary outcomes (pruritus: OR = 3.09, power = 99.96%; fatigue: OR = 0.37, power = 99.73%), exceeding the minimum required sample of 51 patients for 80% power.

For comparisons between individuals with PSC alone and those with PSC and MASLD overlap, the two-sample Wilcoxon rank-sum (Mann–Whitney U) test was used for continuous variables, and Fisher’s exact test was applied for categorical variables. Statistical significance was defined as *p* < 0.05.

To assess the impact of MASLD overlap on fatigue and pruritus intensity, univariate analyses were first conducted to evaluate the unadjusted associations between MASLD and each patient-reported outcome. Subsequently, separate ordinal logistic regression models were developed to independently predict pruritus and fatigue intensity. Both models were adjusted for covariates deemed clinically and statistically significant. Specifically, the pruritus model included sex and bilirubin levels, while the fatigue model included body mass index, diabetes mellitus status, and pruritus intensity. MASLD presence was incorporated as the primary predictor in both multivariate regression models.

All statistical analyses were conducted using Stata version 18.0 (StataCorp LLC, College Station, TX, USA).

## 3. Results

The baseline characteristics of the participants (*n* = 103) were analyzed and are detailed in Table 1. MASLD prevalence was found to be 33%. Individuals with PSC/MASLD tended to be older (median age: 55 vs. 46 years, *p* = 0.006), and exhibited a higher BMI (median: 30 vs. 25, *p* < 0.001) and an increased incidence of comorbidities such as hypertension (38% vs. 14%, *p* = 0.011) and diabetes mellitus (DM) (15% vs. 1%, *p* = 0.014). Small duct involvement was identified in nearly half of individuals with MASLD overlap (48%), whereas 51% of patients without this overlap had large duct involvement (*p* = 0.002). Furthermore, 53% of individuals with PSC/MASLD had no concomitant IBD, in contrast to the PSC-only group, where a higher prevalence of IBD was observed, leaving only 35% without this condition. Interestingly, a history of liver transplantation was documented in 18% of patients with PSC alone, compared to only 3% of those with PSC/MASLD, a difference that approached statistical significance (*p* = 0.055).

**Figure 2:**
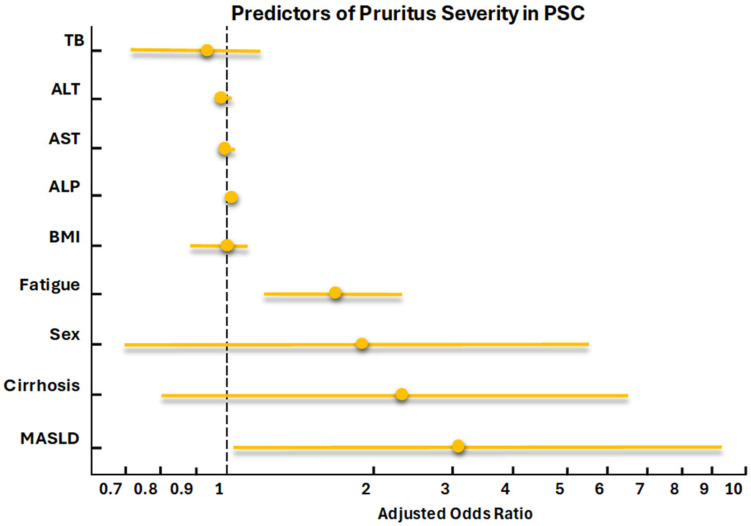
ALP: alkaline phosphatase, ALT: alanine aminotransferase, AST: aspartate aminotransferase, BMI: body mass index, MASLD: Metabolic dysfunction-associated steatotic liver disease, TB: Total bilirubin.

Ordinal logistic regression models revealed the impact of MASLD overlap on the patient-reported outcomes. The model predicting pruritus (Supplementary Table S1) explained approximately 25% of the variability, as indicated by McFadden’s Pseudo R^2^ = 0.2545, and was overall significant (likelihood ratio chi-square 50.27, 9 degrees of freedom, *p* < 0.001). It also demonstrated moderate discriminatory ability (Somers’ D = 0.36, c-index = 0.68), indicating adequate predictive performance. The results evidenced that MASLD overlap in PSC patients was independently associated with increased pruritus severity (OR 3.09, 95% CI 1.02–9.28, *p* = 0.044). Additionally, elevated alkaline phosphatase levels (OR 1.00, 95% CI 1.00–1.008, *p* = 0.003) and higher fatigue scores (OR 1.75, 95% CI 1.28–2.39, *p* < 0.001) were significantly associated with increased pruritus severity (Figure 2).

**Figure 3:**
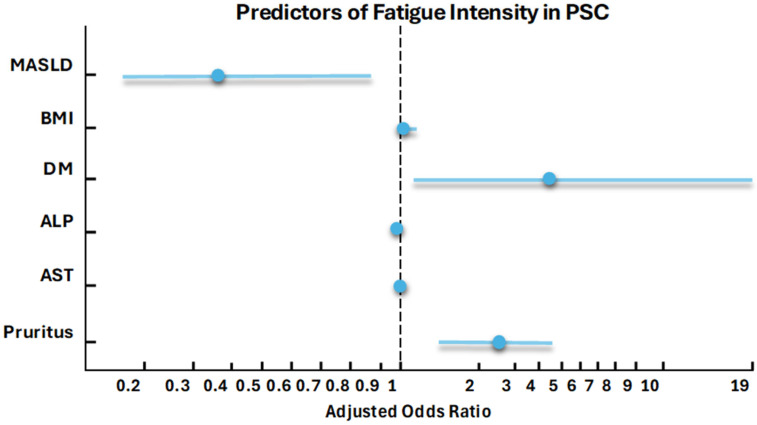
ALP: alkaline phosphatase, AST: aspartate aminotransferase, BMI: body mass index, DM: diabetes mellitus, MASLD: Metabolic dysfunction-associated steatotic liver disease.

In contrast, the multivariate ordinal logistic regression model predicting fatigue intensity (Supplementary Table S2) revealed that MASLD overlap in PSC patients was associated with lower fatigue levels (OR 0.37, 95% CI 0.16–0.85, *p* = 0.020). Conversely, diabetes mellitus (OR 4.48, 95% CI 1.05–19.10, *p* = 0.043) and higher BMI (OR 1.08, 95% CI 1.01–1.16, *p* = 0.018) were linked to increased fatigue intensity. Additionally, pruritus was identified as a strong predictor of fatigue (OR 2.64, 95% CI 1.51–4.61, *p* = 0.001) across both PSC-only and PSC/MASLD patients (Figure 3). This model was statistically significant overall (likelihood ratio chi-square 35.36, 6 degrees of freedom, *p* < 0.001) and demonstrated moderate discriminative ability (Somers’ D = 0.33, c-index = 0.67). However, it explained only 9.27% of the variability in fatigue intensity, according to McFadden’s Pseudo R^2^ = 0.0927, suggesting that other unmeasured factors may contribute substantially to fatigue in this population.

## 4. Discussion

Most studies exploring the coexistence of MASLD with other liver disease have primarily focused on other overlapping etiologies, such as primary biliary cholangitis (PBC), where MASLD appears to worsen patient prognosis [21]. In contrast, individuals with PSC and MASLD overlap remain understudied, and the potential interplay between these two conditions is not well understood. In our cross-sectional analysis, we found a 33% prevalence of concomitant PSC/MASLD, which is more than twice the rate reported in two comparable retrospective studies. The first, conducted in the U.S. in 2014, reported an 8.6% overlap in a cohort of 81 PSC patients [8], while a 2019 Finnish study involving 204 patients found a prevalence of 15% [9]. Several factors may account for this observed increase. One possible explanation is the recent widespread use of Vibration-Controlled Transient Elastography (VCTE), a non-invasive and accessible tool for detecting fibrosis and steatosis, which may improve recognition of MASLD. Additionally, the higher baseline prevalence of MASLD in the U.S. population could contribute to an increased risk of overlap with PSC [22].

Patients with PSC present with a variety of clinical features. In its early stages, most of them are asymptomatic; however, when symptoms do occur, fatigue and pruritus are among the most common reported [6,23,24]. Notably, our study showed that MASLD overlap was associated with increased pruritus intensity, but simultaneously, lower levels of fatigue. To understand this phenomenon, it is important first to consider the subjective nature of symptom reporting, as fatigue and itching are personal, variable experiences best understood through the patient’s lived experience. Beyond the interpretive aspects, biological mechanisms should be considered. While the pathophysiology of pruritus in both PSC and MASLD is not yet fully understood [14], it is believed to involve multiple interacting mechanisms. In cholestatic diseases such as PSC, pruritus results from a combination of factors affecting the biliary tract, including the accumulation of bile acids, bilirubin, lysophosphatidic acid (LPA), and increased endogenous opioids [25]. However, the contribution of these pathways to pruritus in MASLD remains unclear [14]. It is possible that the pro-inflammatory environment characteristic of MASLD may amplify or interact with cholestatic mechanisms, thereby intensifying pruritus when both conditions coexist. Recent evidence supports a broader role for LPA beyond cholestasis. Elevated LPA levels have been linked to hepatic inflammation and fibrosis in MASLD [26]. Recently, He et al. demonstrated that repression of intracellular LPA by GPAM knockdown ameliorated the MASH phenotype in murine models, implicating LPA in MASLD pathogenesis [26]. In this context, patients with concomitant PSC and MASLD may experience more severe pruritus due to the elevated LPA from both disease processes, as seen in our study.

However, although pruritus can disrupt sleep and indirectly exacerbate fatigue [27], we still observed lower fatigue intensity in patients with PSC/MASLD. This paradoxical finding may suggest that the mechanisms driving fatigue and pruritus diverge or interact differently in the context of MASLD overlap. A deeper understanding of these complex symptoms, which significantly impact patients’ quality of life and continue to challenge clinical management, remains essential for improving patient care.

Another important feature of PSC is bile duct involvement. Large duct involvement (ldPSC) is the classical form, representing approximately 89.8% of cases, while small duct PSC (sdPSC) is a rarer variant, occurring in less than 4% of patients [28]. The nature of sdPSC remains unclear; it may represent an early stage of ldPSC, a milder and shorter-duration phenotype, or a distinct clinical entity altogether [29]. Notably, we observed a predominance of sdPSC among patients with MASLD overlap, whereas ldPSC was more common in those with isolated PSC (51%). Given that sdPSC is generally associated with a more benign clinical course, this finding raises the possibility that patients with concomitant PSC and MASLD might have a more favorable long-term prognosis.

PSC is highly associated with inflammatory bowel disease (IBD); approximately 70–85% of patients with PSC have a concurrent diagnosis of IBD, particularly ulcerative colitis (UC), as seen in this cohort [30]. Nevertheless, this association was more evident in patients with PSC alone rather than those with PSC and MASLD, raising the possibility of distinct inflammatory mechanisms underlying each group. In fact, emerging research has shown differential expression of inflammatory microRNAs (miRNAs) in MASLD and IBD, with differences between promoters and regulators of immune response and inflammation [31], perhaps suggesting a different pathogenesis for PSC with MASLD overlap.

Moreover, our analysis found a lower rate of liver transplantation (LT) among PSC/MASLD patients compared to those with isolated PSC, with a trend toward statistical significance (*p* = 0.055). This suggests that the presence of steatosis in MASLD could potentially play a role in altering PSC progression. This observation is clinically relevant, as no disease-modifying therapies have been approved for PSC to date, and liver transplantation remains the only curative option, typically reserved for advanced cases [32,33].

### Limitations

Several limitations merit consideration. The sample was drawn from a single tertiary medical center, which may limit the generalizability of the findings. Participation in this study was voluntary and based on self-reported data, which can introduce both selection and response bias. Furthermore, the symptoms under investigation are inherently subjective, reflecting individual perceptions that can vary substantially among patients, and the surveys rating scales may provide only a partial view of the spectrum of fatigue and pruritus symptoms. We did not as well collect information on other potential contributors to fatigue, such as sleep quality, mood disorders, or concurrent use of medication that could affect pruritus or fatigue and may have influenced symptom reporting. In addition, the presence and severity of MASH, specifically the degree of hepatic fibrosis, were not systematically assessed among participants, which limits our ability to characterize disease severity within the MASLD group. Finally, the cross-sectional design precludes causal inference and does not allow assessment of temporal relationships or disease progression over time. Despite these limitations, this study remains among the few to prioritize patient-reported outcomes in PSC and highlights the clinical relevance of MASLD overlap, an area warranting further investigation through prospective, multi-center studies.

## 5. Conclusions

PSC patients with MASLD overlap exhibit a distinct clinical phenotype. The lower frequency of large-duct involvement, lower rates of IBD and liver transplantation, together with patient-reported lower fatigue intensity, suggest that the coexistence of MASLD in individuals with PSC may reflect distinct metabolic and inflammatory mechanisms that could potentially alter PSC disease progression. However, the paradoxically increased pruritus intensity in this subgroup underscores the complexity of this overlap syndrome and emphasizes the need for further studies to elucidate the underlying pathophysiology, establish causality, and evaluate how this overlap may influence long-term prognosis and optimal management strategies in PSC.

## Figures and Tables

**Table 1:** Baseline clinical characteristics (*n* = 103).

Variable	PSC Only (*n* = 69)	PSC/MASLD (*n* = 34)	*p* Value
Age, (IQR)	46 (35–60)	55 (47–66)	<0.006 *
Sex, *n* (%)			0.835
Female	34 (49)	18 (53)	
Male	35 (51)	16 (47)	
Race/ethnicity, *n* (%)			0.203
White Caucasian	57 (83)	26 (76)	
Black	7 (10)	2 (6)	
Hispanic	1 (1)	4 (12)	
Asian	2 (2)	1 (3)	
BMI, (IQR)	25 (23–28)	30 (26–34)	<0.001 *
Bile Duct Involvement, *n* (%)			0.002 *
Small duct disease	7 (12)	16 (48)	
Large duct disease	30 (51)	10 (30)	
Dominant stricture	17 (29)	5 (15)	
Unknown	5 (8)	2 (6)	
IBD, *n* (%)			0.219
Ulcerative colitis	36 (52)	13 (38)	
Crohn’s disease	9 (13)	3 (9)	
Cirrhosis, *n* (%)	23 (34)	7 (21)	0.249
Cholangiocarcinoma, *n* (%)	1 (1.4)	0	1.000
Liver Transplant, *n* (%)	12 (18)	1 (3)	0.055
Hypertension, *n* (%)	10 (14)	13 (38)	0.011 *
DM, *n* (%)	1 (1)	5 (15)	0.014 *
**Laboratory values**			
ALP, (IQR)	108 (86–250)	125 (92–233)	0.680
ALT, (IQR)	28 (16–59)	31 (22–49)	0.667
AST, (IQR)	29 (20–52)	28 (22–40)	0.735
Total Cholesterol, (IQR)	189 (169–215)	197 (142–231)	0.970
LDL, (IQR)	101 (74–132)	97 (74–133)	0.801
HDL, (IQR)	65 (46–76)	58 (50–83)	0.902
Triglycerides	97 (65–134)	94 (61–163)	0.924
Total Bilirubin, (IQR)	0.6 (0.4–1.1)	0.5 (0.4–0.6)	0.129
Albumin, (IQR)	4.4 (4–4.6)	4.4 (4–4.6)	0.845
WBC–Leukocytes (IQR)	6.2 (4.6–8.1)	7.5 (5.4–8.6)	0.037 *
Platelets, (IQR)	208 (163–286)	268 (220–348)	0.011 *
INR, (IQR)	1.1 (1–1.2)	1.1 (1–1.1)	0.292
Creatinine, (IQR)	0.8 (0.7–0.9)	0.8 (0.7–1)	0.584
**Patient-Reported Outcome**			
**Fatigue Intensity, *n* (%)**			0.566
None of the time	11 (16)	3 (9)	
Hardly any of the time	13 (19)	9 (26)	
A little of the time	13 (19)	9 (26)	
Some of the time	12 (17)	7 (21)	
A good bit of the time	5 (7)	3 (9)	
Most of the time	9 (13)	1 (3)	
All of the time	6 (9)	2 (6)	
**Pruritus, *n* (%)**			0.390
Not present	46 (66)	19 (55)	
Mild	14 (20)	8 (23)	
Moderate	7 (10)	7 (20)	
Unbearable	2 (3)	0 (0)	

* *p* ˂ 0.05. ALP: alkaline phosphatase, ALT: alanine aminotransferase, AST: aspartate aminotransferase, BMI: body mass index, DM: diabetes mellitus, IBD: inflammatory bowel disease, MASLD: Metabolic dysfunction-associated steatotic liver disease.

## Data Availability

The data can be obtained from the corresponding author upon reasonable request.

## Supplementary Materials

The following supporting information can be downloaded at: https://www.mdpi.com/article/10.3390/jcm14228083/s1, Table S1: Ordinal Logistic Regression Model of Pruritus Predictors In PSC; Table S2: Ordinal Logistic Regression Model of Fatigue Predictors In PSC.

## Abbreviations

The following abbreviations are used in this manuscript:

AASLD	American Association for the Study of Liver Diseases
AIH	Autoimmune hepatitis
ALB	Albumin
ALD	Alcohol-related liver disease
ALP	Alkaline phosphatase
ALT	Alanine transaminase
AST	Aspartate aminotransferase
BMI	Body mass index
CLDQ	Chronic Liver Disease Questionnaire
DM	Diabetes mellitus
GPAM	Glycerol-3-phosphate acyltransferase, mitochondrial
IBD	Inflammatory bowel disease
INR	International normalized ratio
IQR	Interquartile ranges
ldPSC	Large duct involvement
LPA	Lysophosphatidic acid
LT	Liver transplantation
MASH	Metabolic dysfunction-associated steatohepatitis
MASLD	Metabolic dysfunction-associated steatotic liver disease
MetALD	Metabolic and alcohol-associated liver disease
miRNAs	MicroRNAs
MRI	Magnetic resonance imaging
PBC	Primary biliary cholangitis
PCC	Patient-centered care
PL	Platelets
PROs	Patient-reported outcome
PSC	Primary sclerosing cholangitis
PT	Prothrombin time
REDCap	Research Electronic Data Capture
sdPSC	Small duct PSC
TB	Total bilirubin
UC	Ulcerative Colitis
VCTE	Vibration-Controlled Transient Elastography
WBC	White blood cell count

## References

[1] Díaz L.A., Lazarus J.V., Fuentes-López E., Idalsoaga F., Ayares G., Desaleng H., Danpanichkul P., Cotter T.G., Dunn W., Barrera F. (2024). Disparities in steatosis prevalence in the United States by race or ethnicity according to the 2023 criteria. Commun. Med..

[2] Miao L., Targher G., Byrne C.D., Cao Y.Y., Zheng M.H. (2024). Current status and future trends of the global burden of MASLD. Trends Endocrinol. Metab..

[3] Luo N., Zhang X., Huang J., Chen H., Tang H. (2024). Prevalence of steatotic liver disease and associated fibrosis in the United States: Results from NHANES 2017–March 2020. J. Hepatol..

[4] Wang D., Xu Y., Zhu Z., Li Y., Li X., Li Y., Shen H., Wu W., Liu Y., Han C. (2022). Changes in the global, regional, and national burdens of NAFLD from 1990 to 2019: A systematic analysis of the Global Burden of Disease Study 2019. Front. Nutr..

[5] Younossi Z.M., Kalligeros M., Henry L. (2025). Epidemiology of metabolic dysfunction-associated steatotic liver disease. Clin. Mol. Hepatol..

[6] Trivedi P.J., Bowlus C.L., Yimam K.K., Razavi H., Estes C. (2022). Epidemiology, natural history, and outcomes of primary sclerosing cholangitis: A systematic review of population-based studies. Clin. Gastroenterol. Hepatol..

[7] Bambha K., Kim W.R., Talwalkar J., Torgerson H., Benson J.T., Therneau T.M., Loftus E.V., Yawn B.P., Dickson E., Melton L. (2003). Incidence, clinical spectrum, and outcomes of primary sclerosing cholangitis in a United States community. Gastroenterology.

[8] Doycheva I., Cox K., Haseeb A., Adler D.G. (2014). Prevalence and relevance of nonalcoholic fatty liver disease in patients with primary sclerosing cholangitis. Dig. Dis. Sci..

[9] Danielsson O., Vesterinen T., Arola J., Åberg F., Nissinen M.J. (2024). Coexistence of metabolic-associated fatty liver disease and autoimmune or toxic liver disease. Eur. J. Gastroenterol. Hepatol..

[10] Kimbell B., Boyd K., Kendall M., Iredale J., Murray S.A. (2015). Managing uncertainty in advanced liver disease: A qualitative, multiperspective, serial interview study. BMJ Open.

[11] Loesken C., Maehder K., Buck L., Hartl J., Löwe B., Schramm C., Toussaint A. (2023). Understanding illness experiences of patients with primary sclerosing cholangitis: A qualitative analysis within the SOMA.LIV study. BMC Gastroenterol..

[12] Swain M.G., Pettersson B., Meyers O., Venerus M., Oscarsson J. (2023). A qualitative patient interview study to understand the experience of patients with nonalcoholic steatohepatitis. Hepatol. Commun..

[13] van Munster K.N., Dijkgraaf M.G.W., Oude Elferink R.P.J., Beuers U., Ponsioen C.Y. (2022). Symptom patterns in the daily life of PSC patients. Liver Int..

[14] Yilmaz Y., Keklikkiran C., Racila A., Stepanova M., Younossi Z.M. (2025). Pruritus and fatigue in patients with metabolic dysfunction–associated steatotic liver disease: A study of Turkish patients from the Global NASH/MASH Registry. Clin. Transl. Gastroenterol..

[15] Royeck S., Mess C., Weigel A., Löwe B., Toussaint A., Schramm C., Mora M.S., Huber T.B., Zeidler C., Witte F. (2025). Comparative analysis of patient-reported outcomes in chronic pruritus: Patients with chronic liver or kidney diseases exhibit higher psychological distress than those with atopic dermatitis. Dermatol. Ther..

[16] Kanagalingam G., Allen J., Chin G.H., Lee H.M. (2025). Palliative care and chronic liver disease: Barriers to care, health disparities and the role of health literacy. Ann. Palliat. Med..

[17] Hansen L., Chang M.F., Hiatt S., Dieckmann N.F., Lee C.S. (2024). Distinct longitudinal trajectories of symptom burden predict clinical outcomes in end-stage liver disease. Clin. Transl. Gastroenterol..

[18] Kim H.P., Lieber S.R., Rogers M.E., Moon A.M., Loiselle M., Walker J., Assis D.N., Safer R., Gomel R., Evon D.M. (2020). A systematic review of patient-reported outcomes in primary biliary cholangitis and primary sclerosing cholangitis. Hepatol. Commun..

[19] Bowlus C.L., Arrivé L., Bergquist A., Deneau M., Forman L., Ilyas S.I., Lunsford K.E., Martinez M., Sapisochin G., Shroff R. (2023). AASLD practice guidance on primary sclerosing cholangitis and cholangiocarcinoma. Hepatology.

[20] Rinella M.E., Lazarus J.V., Ratziu V., Francque S.M., Sanyal A.J., Kanwal F., Romero D., Abdelmalek M.F., Anstee Q.M., Arab J.P. (2023). A multisociety Delphi consensus statement on new fatty liver disease nomenclature. Hepatology.

[21] Hernández-Pérez M., Riado D., Pena E., Méndez C., Pinedo F., Ramos P., Castillo P., Romero M., Fernández-Rodríguez C., Olveira A. (2024). The overlap with metabolic dysfunction-associated steatotic liver disease negatively affects outcomes of primary biliary cholangitis. Aliment. Pharmacol. Ther..

[22] Mehta T.I., Weissman S., Fung B.M., Sotiriadis J., Lindor K.D., Tabibian J.H. (2021). Global incidence, prevalence and features of primary sclerosing cholangitis: A systematic review and meta-analysis. Liver Int..

[23] Sohal A., Kayani S., Kowdley K.V. (2024). Primary Sclerosing Cholangitis: Epidemiology, Diagnosis, and Presentation. Clin. Liver Dis..

[24] Takakura W.R., Tabibian J.H., Bowlus C.L. (2017). The evolution of natural history of primary sclerosing cholangitis. Curr. Opin. Gastroenterol..

[25] Vander Does A., Levy C., Yosipovitch G. (2022). Cholestatic Itch: Our Current Understanding of Pathophysiology and Treatments. Am. J. Clin. Dermatol..

[26] He L., She X., Guo L., Gao M., Wang S., Lu Z., Guo H., Li R., Nie Y., Xing J. (2025). Hepatic AKAP1 deficiency exacerbates diet-induced MASLD by enhancing GPAT1-mediated lysophosphatidic acid synthesis. Nat. Commun..

[27] Patel S.P., Khanna R., Choi J., Williams K.A., Roh Y.S., Hong M.S., Sutaria N.H., Pritchard T., Kwatra M.M., Kwatra S.G. (2021). Sleep disturbance in adults with chronic pruritic dermatoses is associated with increased C-reactive protein levels. J. Am. Acad. Dermatol..

[28] Weismüller T.J., Trivedi P.J., Bergquist A., Imam M., Lenzen H., Ponsioen C.Y., Holm K., Gotthardt D., Färkkilä M.A., Marschall H.-U. (2017). Patient Age, Sex, and Inflammatory Bowel Disease Phenotype Associate With Course of Primary Sclerosing Cholangitis. Gastroenterology.

[29] Nguyen C.M., Kline K.T., Stevenson H.L., Khan K., Parupudi S. (2022). Small duct primary sclerosing cholangitis: A discrete variant or a bridge to large duct disease, a practical review. World J. Hepatol..

[30] Kim Y.S., Hurley E.H., Park Y., Ko S. (2023). Primary sclerosing cholangitis (PSC) and inflammatory bowel disease (IBD): A condition exemplifying the crosstalk of the gut-liver axis. Exp. Mol. Med..

[31] Popa M.L., Ichim C., Anderco P., Todor S.B., Pop-Lodromanean D. (2025). MicroRNAs in the Diagnosis of Digestive Diseases: A Comprehensive Review. J. Clin. Med..

[32] Hasegawa S., Yoneda M., Kurita Y., Nogami A., Honda Y., Hosono K., Nakajima A. (2021). Cholestatic Liver Disease: Current Treatment Strategies and New Therapeutic Agents. Drugs.

[33] Björnsson E.S., Kalaitzakis E. (2021). Recent advances in the treatment of primary sclerosing cholangitis. Expert. Rev. Gastroenterol. Hepatol..

