# Supplementary material for: Impact of Metabolic Dysfunction-Associated Steatotic Liver Disease on Fatigue and Pruritus in Primary Sclerosing Cholangitis: A U.S. Single-Center Study
Source: J Clin Med. 2025 Nov 14;14(22):8083. doi: 10.3390/jcm14228083 (PMC12653078; doi:10.3390/jcm14228083)
Supplement: Supplementary file 1 [file jcm-14-08083-s001.zip › jcm-3934383-supplementary.pdf]

Supplementary Table S1. Ordinal Logistic Regression Model Of Pruritus Predictors In PSC.

	Univariate			Multivariate		
	**Odds Ratio**	**95% CI**	**p Value**	**Odds Ratio**	**95% CI**	**p Value**
MASLD	1.56	0.69–3.53	0.278	3.09	1.02–9.28	0.044*
Age	1.00	0.98–1.03	0.680			
Sex	1.63	0.73–3.60	0.227	1.94	0.68–5.46	0.210
Race	0.71	0.38–1.32	0.286			
BMI	1.07	1.00–1.14	0.029*	1.00	0.92–1.10	0.879
Bile Duct Involvement	1.02	0.65–1.62	0.903			
IBD	0.96	0.53–1.71	0.891			
Cirrhosis	2.39	1.02–5.56	0.043*	2.28	0.80–6.53	0.122
Liver Transplant	0.69	0.20–2.39	0.568			
Hypertension	1.03	0.41–2.59	0.938			
DM	2.04	0.41–9.94	0.376			
Alkaline phosphatase	1.00	1.00–1.008	<0.001*	1.01	1.00–1.008	0.003*
ALT	1.01	1.00–1.02	0.001*	0.99	0.97–1.02	0.825
AST	1.01	1.00–1.03	<0.001*	1.00	0.98–1.03	0.394
Total Cholesterol	0.99	0.98–1.00	0.913			
LDL	1.01	0.99–1.02	0.131			
HDL	0.98	0.96–1.00	0.290			
Triglycerides	0.99	0.98–1.00	0.399			
Total Bilirubin	1.23	0.92–1.65	0.148	0.93	0.72–1.20	0.599
Albumin	0.57	0.24–1.38	0.220			
WBC-Leukocytes	1.04	0.92–1.18	0.463			
Platelets	1.00	0.99–1.00	0.679			
INR	1.17	0.59–2.28	0.644			
Creatinine	0.24	0.03–1.65	0.148			
Fatigue	1.76	1.37–2.27	<0.001*	1.75	1.28–2.39	<0.001*

*$p < 0.05$. ALT: alanine aminotransferase, AST: aspartate aminotransferase, BMI: body mass index, DM: diabetes mellitus, IBD: inflammatory bowel disease, MASLD: Metabolic dysfunction-associated steatotic liver disease.

Supplementary Table S2. Ordinal Logistic Regression Model Of Fatigue Predictors In PSC.

	Univariate			Multivariate		
	**Odds Ratio**	**95% CI**	**p Value**	**Odds Ratio**	**95% CI**	**p Value**
MASLD	0.81	0.40–1.66	0.582	0.37	0.16–0.85	0.020*
Age	0.99	0.97–1.01	0.813			
Gender	1.83	0.91–3.65	0.087			
Race	1.06	0.67–1.66	0.796			
BMI	1.08	1.01–1.15	0.012*	1.08	1.01–1.16	0.018*
Bile Duct Involvement	0.90	0.60–1.35	0.641			
IBD	0.89	0.54–1.47	0.659			
Cirrhosis	1.31	0.61–2.82	0.476			
Liver Transplant	1.16	0.41–3.25	0.774			
Hypertension	1.33	0.60–2.95	0.477			
DM	3.95	1.00–15.49	0.048*	4.48	1.05–19.10	0.043*
Alkaline phosphatase	1.00	1.00–1.004	0.026*	0.99	0.99–1.00	0.906
ALT	1.00	0.99–1.01	0.118			
AST	1.01	1.00–1.02	0.008*	1.00	0.99–1.02	0.350
Total Cholesterol	1.00	0.99–1.01	0.755			
LDL	1.00	0.99–1.01	0.234			
HDL	0.99	0.98–1.01	0.794			
Triglycerides	0.99	0.98–1.00	0.080			
Total Bilirubin	1.21	0.98–1.49	0.062			
Albumin	0.54	0.25–1.18	0.125			
WBC - Leukocytes	1.07	0.95–1.19	0.230			
Platelets	1.00	0.99–1.00	0.715			
INR	1.39	0.72–2.71	0.319			
Creatinine	0.41	0.08–2.05	0.283			
Pruritus	3.11	1.93–5.02	<0.001*	2.64	1.51–4.61	0.001*

*$p < 0.05$. ALT: alanine aminotransferase, AST: aspartate aminotransferase, BMI: body mass index, DM: diabetes mellitus, IBD: inflammatory bowel disease, MASLD: Metabolic dysfunction-associated steatotic liver disease.
